# Advanced Biomaterial-Based In Vitro Osteoarthritis Models: Integrating Sex as a Biological Variable in Hormonal, Subchondral Bone, and Mechanobiological Pathways

**DOI:** 10.3390/jfb17010035

**Published:** 2026-01-10

**Authors:** Elisa Capuana, Angela De Luca, Viviana Costa, Lavinia Raimondi, Daniele Bellavia, Valerio Brucato, Gianluca Giavaresi, Vincenzo La Carrubba

**Affiliations:** 1 Department of Engineering, University of Palermo, Viale delle Scienze, Ed. 8, 90128 Palermo, Italy; elisa.capuana@unipa.it (E.C.); valerio.brucato@unipa.it (V.B.); vincenzo.lacarrubba@unipa.it (V.L.C.); 2 CS-Surgical Sciences and Technologies-SS Omics Science Platform for Personalized Orthopedics, IRCCS Istituto Ortopedico Rizzoli, 40136 Bologna, Italy; viviana.costa@ior.it (V.C.); lavinia.raimondi@ior.it (L.R.); daniele.bellavia@ior.it (D.B.); gianluca.giavaresi@ior.it (G.G.)

**Keywords:** osteoarthritis, sex differences, mechanobiology, estrogens, subchondral bone, advanced biomaterials, bone tissue engineering, microfluidic devices, bioreactors

## Abstract

Osteoarthritis (OA) is the most common form of arthritis and represents a major clinical and socioeconomic burden. Epidemiological data consistently show that OA affects women more frequently and, in several joints, more severely than men. Nevertheless, current in vitro models rarely consider sex-specific variables, limiting their ability to capture the biological mechanisms that shape the pathogenesis and progression of OA. Increasing evidence indicates that age-related hormonal fluctuations and subchondral bone remodeling strongly influence OA evolution, and that these processes differ between the sexes. For instance, the decline in estrogen levels during menopause has been associated with accelerated cartilage degeneration, increased osteoclastic activity, and a higher susceptibility to subchondral bone alterations, which may contribute to more aggressive clinical manifestations in women. These mechanisms are only partially reproduced in widely used experimental systems, including traditional biomaterial scaffolds and simplified osteochondral constructs, leaving important sex-dependent pathways unresolved. While advanced biomaterials enable precise control of stiffness, porosity, and biochemical cues, most current in vitro OA models still rely on sex-neutral design assumptions, limiting their ability to reproduce the divergent disease trajectories observed in men and women. By integrating material properties with dynamic loading and tunable hormonal conditions, next-generation in vitro systems could improve mechanistic understanding, increase the reliability of drug screening, and better support the development of sex-specific therapies through the combined efforts of bioengineering, materials science, cell biology, and translational medicine.

## 1. Introduction

Osteoarthritis (OA) is the most prevalent form of arthritis, affecting over 500 million people worldwide [1]. The disease disproportionately affects women, particularly after menopause, who often experience more severe symptoms and a lower quality of life than men [2]. Despite this evidence, osteoarthritis research and therapy development remain largely sex-neutral, reflecting a deeper conceptual assumption that sex acts as a secondary modifier rather than a primary organizer of joint biology. This assumption has profoundly shaped both experimental research and the design and interpretation of in vitro models, leading OA to be widely studied as a sex-neutral condition despite well-documented biological differences between men and women [3,4].

Conventional in vitro models, including two-dimensional (2D) chondrocyte cultures and three-dimensional (3D) models of mechanical damage, often neglect critical factors such as hormone concentrations and subchondral bone remodeling, despite their established relevance in disease onset and progression [5,6,7]. As a result, widely recommended interventions, such as exercise, weight loss, and physical therapy, are commonly applied uniformly, without accounting for the biological differences that influence OA development [8].

Recent studies have highlighted the important role of hormonal regulation, particularly estrogen, in OA pathophysiology. For example, Liao et al. demonstrated that estrogen modulates the production of pro-inflammatory cytokines in human chondrocytes (C28/I2 cell line), and estrogen deficiency has been associated with cartilage degradation by autophagy dysregulation and inflammasome activation mediated by the estrogen receptor [9]. However, conclusions drawn from estrogen-driven inflammatory signaling in isolated chondrocyte cultures are not always consistent with findings from mechanically loaded cartilage or osteochondral models, indicating that endocrine effects are highly context-dependent. Clinically, postmenopausal women show a more rapid OA onset, accompanied by subchondral tissue alterations, including increased osteoclast activity and a higher incidence of subchondral cysts. These alterations may contribute to joint instability and more severe symptoms [10,11].

Therapeutic response may also differ by sex. Non-steroidal anti-inflammatory drugs (NSAIDs), which inhibit cyclooxygenase-2 (COX-2), can be influenced by estrogen, which normally modulates inflammatory pathways [11,12]. Before menopause, this regulation may enhance the response to NSAID, whereas postmenopausal estrogen loss reduces this modulatory effect, potentially diminishing drug efficacy in women [12,13]. OA is frequently associated with comorbidities such as cardiovascular disease, diabetes, hypertension, and depression, further complicating management and increasing socioeconomic impact. Notably, 54.6% of women with OA have at least one comorbidity, and the risk of having three or more is 94% higher than in men [12]. With aging populations and rising female life expectancy, the prevalence and burden of OA are expected to increase further [14]. Addressing these challenges requires a shift from sex-neutral in vitro platforms toward models in which sex-dependent endocrine and biomechanical logic is embedded at the design level, rather than added post hoc. While two-dimensional 2D cultures can provide insights into hormone effects by adding sex-specific factors to the medium, they cannot reproduce the extracellular matrix (ECM) environment or joint biomechanics. Three-dimensional (3D) systems, such as biomaterial-based scaffolds, bioreactors, and microfluidic platforms, better replicate tissue architecture and integrate mechanical and molecular cues, allowing the study of complex interactions between hormonal signaling, biomechanical loading, and ECM organization [15,16]. In this context, scaffold-based biomaterial systems have become central to in vitro OA modeling, as they enable the reconstruction of osteochondral architecture and mechanical properties with increasing fidelity. However, current scaffold materials are predominantly designed to reproduce generic tissue stiffness, porosity, and composition, while critical limitations persist. In particular, most scaffolds are implemented under static or simplified conditions, lack integration of dynamic hormonal exposure, and are rarely evaluated for sex-dependent differences in matrix deposition, mineralization kinetics, or mechanotransductive signaling. As a result, scaffold-based models often reproduce structural features of the osteochondral unit while failing to capture the biological asymmetries that characterize OA progression in men and women.

Building on this perspective, this review examines the sex-specific mechanisms regulating OA, with particular attention to hormonal influences and subchondral bone remodeling. While osteoarthritis is a whole-joint disease involving cartilage, synovium, and bone, this review deliberately emphasizes subchondral bone as a central mechanobiological and hormone-responsive compartment, supported by growing evidence that early subchondral remodeling drives cartilage degeneration through osteochondral crosstalk in a sex-dependent manner. This review then evaluates how these mechanisms are (or are not) captured by current experimental systems, including emerging biomaterial-enabled platforms, highlighting critical gaps that constrain translational research. Finally, it discusses next-generation in vitro approaches aimed at advancing personalized, sex-specific therapeutic strategies for OA, arguing that sex should be embedded as a system-level design principle, rather than introduced as an experimental variable, in the development of osteoarthritis models.

## 2. Sex Differences in Osteoarthritis: Hormonal Influences, Subchondral Bone Remodeling, and Response to Therapy

### 2.1. The Role of Hormones in the Pathophysiology of OA

Although OA has been widely investigated, most mechanistic studies have traditionally emphasized biomolecular and biomechanical mechanisms, while sex-related variables have received comparatively limited attention [17,18]. However, clinical and experimental evidence consistently indicates that the higher susceptibility of postmenopausal women to OA is closely associated with declining estrogen levels. From a quantitative perspective, these effects occur against a background of profound endocrine changes. Circulating estradiol levels in premenopausal women typically range from ~20 to 400 pg/mL depending on menstrual cycle phase, whereas postmenopausal concentrations generally decline to <30 pg/mL, representing an order-of-magnitude reduction in estrogenic exposure [19,20]. Estrogen loss does not simply modulate cartilage catabolism, but fundamentally alters how joint tissues perceive and respond to mechanical stimuli, redefining the mechanobiological setpoint of the osteochondral unit [21,22].

**Figure 1:**
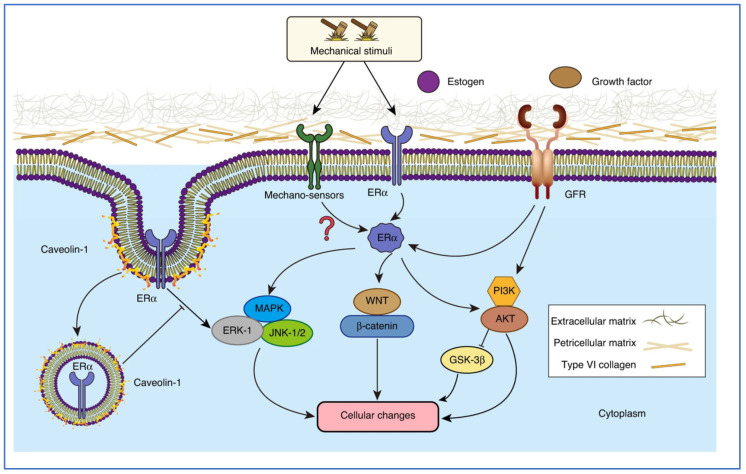
The role of estrogen in maintaining subchondral bone integrity: this model highlights the distinct pathways through which estrogen deficiency may accelerate OA progression, linking hormonal fluctuations to biomechanical deterioration. ERα: estrogen receptor α, MAPK: mitogen-activated protein kinase, ERK: extracellular signal-regulated kinase, JNK: c-Jun N-terminal kinase, GFR: growth factor receptor, PI3K: phosphatidylinositol-3-kinase, AKT: protein kinase B, and GSK-3β: glycogen synthase kinase 3 beta. Reprinted from Ref. [28].

At the tissue level, these hormonal effects are mediated through estrogen receptors ER-α and ER-β are highly expressed in joint tissues and exert distinct regulatory effects on cartilage turnover, bone remodeling, and inflammation [23,24]. ER-α, predominantly expressed in chondrocytes, influences cell proliferation and survival, whereas ER-β, detected in osteoblasts and synovial cells, modulates bone homeostasis and cytokine production [25]. As illustrated in Figure 1, ER-α regulates the mitogen-activated protein kinase (MAPK) pathway through rapid, non-genomic mechanisms involving membrane-associated ER-α and caveolin-1, which integrate mechanical stimuli and growth factor signaling [26]. At physiological estrogen levels, ER-α contributes to balanced activation of the extracellular signal-regulated kinase 1/2 (ERK1/2) and c-Jun N-terminal kinase (JNK) pathways, keeping inflammatory signaling and extracellular matrix turnover under control. Under conditions of estrogenic deficiency, this regulatory control is partially lost, leading to aberrant MAPK activation in response to mechanical stress and growth factors. Although the precise molecular mechanisms underlying this dysregulation are not yet fully understood, evidence suggests that reduced ER-α signaling leads to increased MAPK activity, increased expression of matrix metalloproteinases, and accelerated extracellular matrix degradation, thereby promoting OA progression [27,28].

**Figure 2:**
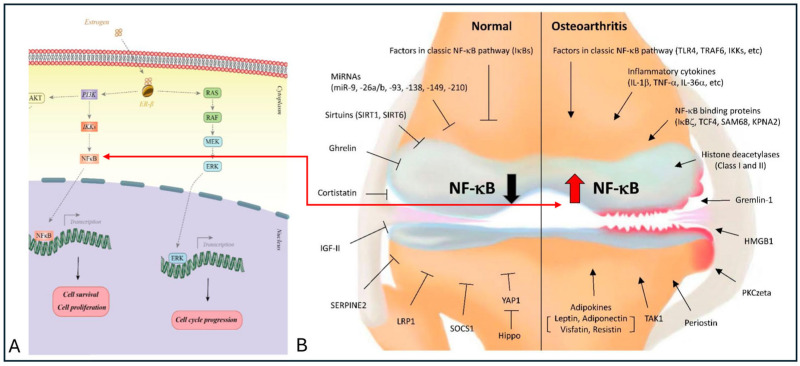
ERβ modulation of NF-κB signaling: (**A**) Estrogen binds to ERβ, influencing the NF-κB pathway and supporting cell cycle progression, survival, and proliferation under normal conditions [31]. (**B**) Reduced estrogen levels in osteoarthritis alter NF-κB activity, promoting cartilage breakdown and bone changes. Reprinted from Ref. [32].

Figure 2 highlights the role of ER-β in modulating the nuclear factor kappa-light-chain-enhancer of activated B cells (NF-κB) pathway [29]. Under physiological estrogen levels, ER-β activation suppresses NF-κB signaling, thereby limiting the expression of pro-inflammatory cytokines and catabolic mediators involved in cartilage and subchondral bone homeostasis. In osteoarthritic conditions, reduced estrogen levels lead to diminished ER-β signaling, resulting in enhanced NF-κB activity and sustained inflammatory responses [30]. Together with ER-α-mediated alterations in mechanotransduction pathways, the loss of ER-β-dependent control over NF-κB help explain why menopause markedly accelerates both structural and inflammatory changes characteristic of OA. However, these findings also reveal a broader limitation of the current literature: estrogen signaling has been predominantly examined through isolated molecular pathways, with limited integration of its role in regulating tissue-scale mechanobiological responses across experimental models. While ERα- and ERβ-mediated pathways are often discussed separately in the literature, few experimental studies directly compare their relative contribution under combined hormonal and mechanical stimulation, limiting cross-study interpretability.

In men, OA usually develops later in life and is often associated with mechanical overload, trauma, or metabolic dysregulation rather than endocrine changes [10]. Nonetheless, male hormones, particularly testosterone, also influence cartilage metabolism and bone density, although evidence remains inconsistent [33,34]. Large population-based studies, including NHANES (National Health and Nutrition Examination Survey), indicate that hypogonadism increases OA risk, but primarily when testosterone falls below a critical physiological threshold [35]. The discrepancies observed between population-based, cellular, and animal studies suggest that androgen-related effects in OA cannot be interpreted in isolation, but rather emerge from interactions with mechanical loading, metabolic status, and local aromatization dynamics. Mechanistically, reduced aromatization of testosterone into estradiol may diminish ER-α/ER-β activation in cartilage and reduces chondroprotection. Testosterone also acts through the androgen receptor (AR), which contributes to osteoblast function and cartilage matrix maintenance [36]. Moreover, testosterone can interact with Wnt/β-catenin signaling, a pathway involved in chondrocyte differentiation and joint homeostasis [37,38]. Lifestyle and metabolic factors further modulate this relationship, complicating the attribution of a purely beneficial or detrimental role to testosterone [39]. In this context, studies reporting protective effects of testosterone on cartilage matrix contrast with clinical observations in aging men, suggesting that hormonal signaling alone is insufficient to explain OA susceptibility without accounting for mechanical and metabolic cofactors.

It is worth noting that women also experience a progressive decline in circulating testosterone levels after menopause, which may contribute to joint tissue alterations in addition to estrogen deficiency [14]. However, the specific role of reduced androgen signaling in postmenopausal OA remains poorly understood and warrants further investigation [34]. Longitudinal studies are therefore required to disentangle direct hormonal effects from metabolic confounders.

Table 1 provides a comparative overview of the sex-specific hormonal mechanisms discussed above, highlighting how estrogen and testosterone differentially regulate joint homeostasis through distinct receptor-mediated pathways and downstream signaling cascades in women and men, and how these differences may contribute to sex-specific OA phenotypes.

### 2.2. The Role of Subchondral Bone in the Pathophysiology of OA

#### 2.2.1. Subchondral Bone Remodeling in OA Progression

Subchondral bone remodeling plays a central role in the pathogenesis of OA and may precede detectable cartilage degeneration. Early alterations, such as increased bone turnover, sclerosis, and microfractures, compromise the mechanical integrity of the joint, elevating stress on the overlying cartilage and promoting matrix degradation [40,41].

**Figure 3:**
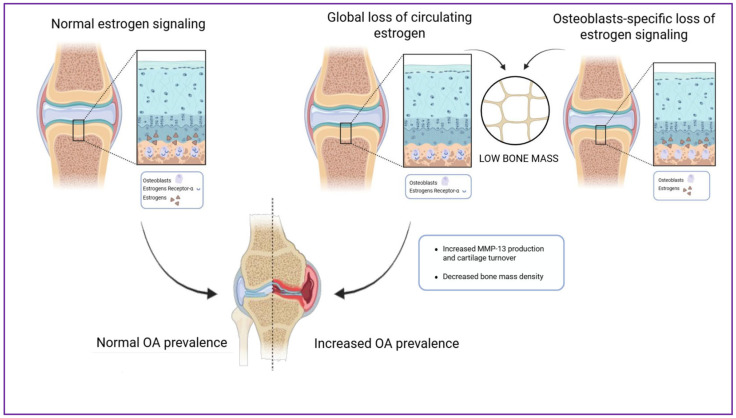
Impact of estrogen signaling on subchondral bone and OA progression. The figure illustrates how estrogen maintains bone-cartilage homeostasis under mechanical loading and contrasts the effects of global estrogen depletion (i.e., menopause) with osteoblast-specific estrogen receptor loss, both of which accelerate subchondral bone loss and OA severity. Although osteoarthritis is often associated with increased bone mineral density and subchondral sclerosis in early disease stages, estrogen deficiency in postmenopausal women is linked to altered bone remodeling, architectural deterioration, and localized bone loss, which are the processes illustrated in this figure.

Sex differences substantially influence how these processes develop. Estrogen normally helps maintain bone homeostasis by stimulating osteoblast activity, inhibiting osteoclast function, and preserving matrix quality [42,43,44]. In menopausal women, estrogen depletion accelerates bone mineral density (BMD) loss and disrupts subchondral architecture (Figure 3).

From a quantitative standpoint, this endocrine shift is accompanied by measurable skeletal changes; longitudinal studies report an average reduction in bone mineral density of 5–10% across the menopausal transition, together with site-specific alterations in subchondral bone microarchitecture [45,46]. Mechanistically, estrogen deficiency enhances osteoclast differentiation and activity through dysregulation of the receptor activator of nuclear factor κB ligand (RANKL)/osteoprotegerin (OPG) signaling axis, thereby shifting bone remodeling toward increased resorption [47,48].

In addition to changes in bone turnover, estrogen deficiency also affects the quality of the subchondral bone matrix. Type I collagen, the main organic component of the bone matrix, actively regulates mineral deposition by providing binding sites for calcium ions and guiding hydroxyapatite nucleation. Recent studies of materials used in living things have shown that changing how collagen and calcium work together can control how much calcium is added to a place, showing that collagen is an active controller of the build-up of calcium, not just a support for it [49].

In several studies, advanced imaging techniques, including micro-CT and nanoindentation, have shown that women experience greater reductions in subchondral bone stiffness than men, which correlates with higher pain and disability scores [50]. While imaging and biomarker studies consistently report sex-dependent alterations in subchondral remodeling, they rarely converge on a unified mechanistic framework capable of explaining how early bone changes causally drive cartilage degeneration across sexes. In men, subchondral bone changes are more frequently associated with chronic mechanical loading. Although greater muscle mass has been proposed in biomechanical studies to provide partial protection by attenuating joint stress, once bone stiffness declines (particularly in later disease stages), men follow a progression trajectory comparable to that observed in women [51,52]. By contrast, the earlier onset of OA in women may reflect menopause-associated changes in body composition, including reduced muscle mass and increased fat mass, which diminish biomechanical protection and increase joint loading, thereby accelerating disease initiation [53].

#### 2.2.2. Biomarkers for Bone Remodeling in OA

Given the centrality of bone remodeling in OA, several biomarkers have emerged as promising tools to monitor disease-associated changes in tissue turnover [54,55]. N-terminal pro-collagen type I (PINP) reflects bone formation, whereas C-terminal telopeptide of collagen type I (CTX-I) indicates bone resorption [56]. CTX-II, derived from type II collagen degradation, additionally reflects cartilage breakdown [55].

Postmenopausal women often show decreased PINP and elevated CTX-I/CTX-II levels, a pattern associated with enhanced osteoclastic activity and increased cartilage turnover. These trends are well documented in postmenopausal physiology and have also been reported in OA cohorts [57,58]. Interventions such as hormone replacement therapy (HRT) or antiresorptive agents have been shown to reduce CTX levels and may attenuate tissue degeneration in some studies [59,60], although results across clinical trials remain heterogeneous.

Because these biomarkers reflect global bone and cartilage metabolism (and are influenced by conditions such as osteoporosis, systemic inflammation, and fracture healing), their diagnostic and prognostic value in OA is limited [58]. More physiologically experimental systems, particularly sex-specific in vitro models, are needed to clarify the molecular relationships between hormonal signaling, bone remodeling, and cartilage deterioration.

### 2.3. Sex Differences in Response to OA Treatments

Most OA treatments derive from sex-neutral preclinical and clinical evidence, despite clear biological differences in disease mechanisms. Animal models rarely replicate age-related estrogen or testosterone decline, and clinical trials seldom stratify outcomes by sex [18,61,62]. This limits the ability to detect sex-specific therapeutic responses and to design tailored strategies.

In men, clinical management often prioritizes mechanical contributors to disease progression, including weight reduction, physical therapy, and optimization of joint loading [63]. While hormone-based interventions are not routinely considered in male OA, studies in postmenopausal women have reported that combining HRT with exercise may improve pain and function, although evidence remains inconclusive and safety concerns persist [64,65]. For example, HRT has been associated with increased rates of periprosthetic joint infection in some postoperative cohorts [66,67]. In women, therapeutic approaches more directly address hormonal status, and combined HRT–NSAID regimens have been reported to improve symptoms and inflammatory profiles in the short term (3–6 months) [14,68,69], although potential risks, including breast cancer, cardiovascular disease, and stroke, require careful individual evaluation [70].

Selective estrogen receptor modulators (SERMs), such as raloxifene and bazedoxifene, act on estrogen receptors ER-α and ER-β and can improve bone density while potentially exerting protective effects on cartilage in preclinical studies [9,71,72]. In men, SERMs have primarily been studied in the context of prostate cancer and infertility, where they preserve bone density [73,74], but their relevance to OA remains largely unexplored.

These diverging therapeutic responses underscore the need for advanced preclinical platforms that incorporate hormonal signaling, mechanical stimuli, and sex-specific biological variables. Such systems are essential for clarifying why treatments, such as HRT, testosterone replacement therapy (TRT), or SERMs, may be beneficial in some patients yet ineffective or risky in others.

## 3. Current In Vitro Models of OA

### 3.1. From 2D to 3D In Vitro Models

To understand how hormonal cues and subchondral remodeling shape osteoarthritis differently in men and women, in vitro systems must overcome the intrinsic constraints of traditional models. Two-dimensional cultures remain valuable for dissecting individual pathways, such as ERα/β signaling or cytokine-induced catabolism, but their simplified geometry prevents the reproduction of the spatial gradients that mediate sex-specific interactions between cartilage, bone, and synovium [75]. In particular, they cannot replicate the microenvironmental differences through which estrogen decline accelerates cartilage degeneration in women or through which androgen fluctuations modulate osteoblastic activity in men.

Animal models provide a broader physiological context, yet fundamental interspecies differences in cartilage thickness, gait mechanics, and endocrine aging limit their capacity to reproduce human sex-specific trajectories [76,77]. Rodents, for example, do not undergo a menopausal transition comparable to humans, nor do they exhibit the gradual androgen reduction observed in aging men [78]. Consequently, sex-linked alterations in mechanosensitivity, inflammatory priming, or subchondral bone turnover remain difficult to isolate in vivo.

These limitations have accelerated the shift toward controlled 3D platforms, such as biomaterial-based scaffolds, bioreactors, and microfluidic devices, which better emulate the structural, mechanical, and biochemical diversity of the joint environment [79,80]. Crucially, they enable the systematic introduction of sex-associated variables such as hormone levels, receptor expression, and differential mechanotransduction [81,82]. This opens the way for models capable of reproducing not only the general physiology of the osteochondral unit but also its sexually dimorphic behavior across life stages.

### 3.2. Scaffold-Based Strategies for Osteochondral Regeneration

Scaffold-based systems, defined as three-dimensional biomaterial constructs designed to support cell adhesion, organization, and tissue-specific function, offer a versatile framework for reconstructing the osteochondral interface and are well positioned for investigating how sex influences tissue homeostasis and degeneration [83,84].

A broad range of manufacturing techniques has been applied to osteochondral scaffold fabrication [85]. Techniques such as electrospinning and 3D bioprinting enable precise control over fiber alignment, stiffness, and biochemical functionalization, which are parameters closely tied to sex-dependent mechanobiology [86,87]. For example, electrospun PCL (Poly(ε-caprolactone)) nanofibers, often loaded with bioactive molecules, have been shown to induce MSC (mesenchymal stem cells) chondrogenesis [88,89], while bioprinted cell-laden bioinks have supported the fabrication of multiphasic osteochondral constructs [90]. Despite their architectural sophistication, most scaffold-based osteochondral models remain donor-sex–neutral, reflecting persistent limitations at both the structural and biological levels. From a structural perspective, osteochondral scaffolds are typically engineered to reproduce averaged mechanical properties of cartilage and subchondral bone, thereby neglecting well-documented sex-dependent differences in tissue stiffness, anisotropy, and load distribution [91]. As a consequence, scaffold elasticity and interfacial gradients are commonly tuned to generic physiological ranges that may be mechanically permissive for one sex while proving maladaptive for the other [92].

At the biological level, scaffold-based systems frequently decouple matrix architecture from endocrine regulation [93]. Although bioactive coatings and growth factor incorporation are increasingly used to promote osteochondral differentiation, hormonal signaling is generally treated as an external and uniform culture condition rather than as an intrinsic design constraint [94]. This separation limits the ability of scaffolds to reproduce sex-specific feedback loops linking extracellular matrix composition, receptor-mediated signaling (ERα/ERβ or AR), and mechanotransduction pathways that govern tissue remodeling during OA progression. Consequently, these models implicitly assume that extracellular matrix deposition, mineralization kinetics, and mechanotransductive responses are invariant across sexes, which is an assumption that directly contradicts clinical and biological evidence. This sex-neutral design constrains the interpretation of intrinsic differences in ERα/β or AR signaling, differential ECM deposition, and variable mineralization behaviors that may underlie the accelerated subchondral deterioration observed in postmenopausal women compared with age-matched men. Without sex-stratified experimental designs incorporating male- and female-derived cells, controlled hormonal environments, and separate analysis of mechanobiological and matrix-related outcomes, scaffold-based OA models cannot be considered mechanistically interpretable for studying disease progression or therapeutic response [95] These limitations have direct implications for translational reliability. Scaffolds optimized under sex-neutral assumptions may faithfully reproduce matrix deposition or mineralization in vitro yet fail to predict divergent disease trajectories or treatment responses observed clinically between men and women. In particular, hormone-independent degradation kinetics and static material properties are poorly aligned with the temporally dynamic endocrine environments associated with aging, menopause, or hypogonadism. As a result, sex bias is not introduced solely through cell sourcing or experimental readouts but is embedded at the level of material design itself [96,97].

**Figure 4:**
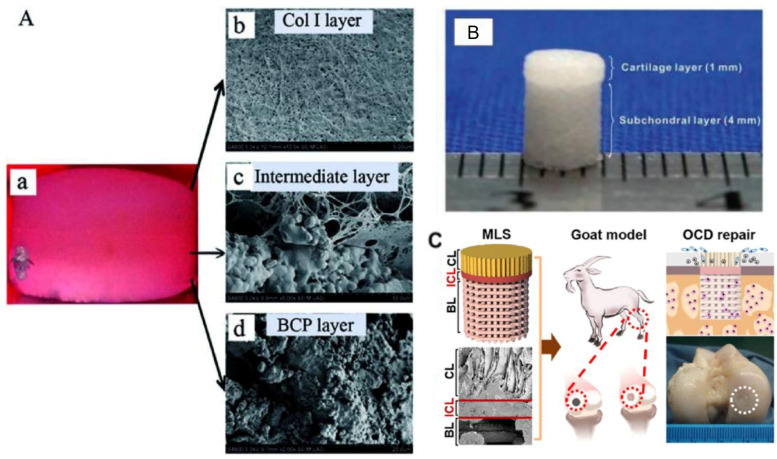
Scaffold architectures designed to replicate the structural complexity of osteochondral tissue: (**A**) tri-layer scaffold (**a**) of type I collagen (Col I) (**b**) combined with an intermediate layer (**c**) and biphasic calcium phosphate (BCP) ceramics (**d**), Reprinted from Ref. [100]. (**B**) bilayered PLGA systems, Reprinted from Ref. [101], and (**C**) hybrid constructs integrating printed bone, interfacial, and cartilage layers. These designs illustrate different approaches to improving osteochondral integration and regeneration, emphasizing biomaterial selection and stratification strategies. Reprinted with permission from Ref. [102], Copyright 2018, American Chemical Society.

Biomaterial selection further influences scaffold performance and reflects a persistent trade-off between mechanical robustness and biological functionality. Multilayer or multiphasic designs emulate the zonal complexity of the osteochondral unit and are theoretically required to model the distinct remodeling patterns observed between sexes [98,99]. Examples include collagen–BCP (biphasic calcium phosphate) trilayers (Figure 4A) [100], bilayered PLGA constructs (Figure 4B) [101], and hybrid 3D-printed architectures incorporating bone, interfacial, and cartilage layers (Figure 4C) [102]. However, their evaluation typically occurs under static or simplified conditions that omit sex-relevant cues such as fluctuating hormone levels, sex-specific loading regimes, or receptor-level mechanotransductive readouts. This disconnect reflects a broader trend in the literature, where advances in scaffold architecture and material design have outpaced the biological interpretation of sex-specific signaling, leading to structurally sophisticated but mechanistically under-informative models.

For these systems to reach their full potential in sex-centered OA modeling, scaffold design must be coupled with controlled endocrine exposure, sex-stratified donor cells, and quantitative metrics capable of capturing sex-dependent responses in cartilage and bone [103].

### 3.3. Bioreactor-Based Approaches for OA Modeling

Bioreactors, defined as engineered culture systems designed to apply controlled physical, mechanical, and biochemical stimuli to living cells or tissues under dynamic conditions, offer controlled, dynamic environments that are indispensable for investigating how sex modulates the mechanobiological pathways involved in osteoarthritis. Mechanical forces, such as compression, hydrostatic pressure, and shear stress, interact with sex hormones and their receptors, producing divergent cellular responses that static cultures cannot capture [104]. Most bioreactor systems apply standardized loading schemes that ignore well-documented sex-dependent differences in joint kinematics. Consequently, these platforms systematically erase the biomechanical conditions under which sex-specific OA phenotypes emerge [103,105].

Dynamic compression remains the most common stimulus. Frequencies between 0.1 and 1 Hz mimic the mechanical patterns associated with daily locomotion and generally enhance ECM synthesis compared with static conditions [106]. However, these stimuli rarely incorporate variables known to differ between men and women, such as increased valgus alignment and altered lateral force transmission in women or higher quadriceps-driven compressive forces in men [107,108]. By ignoring these biomechanical asymmetries, current bioreactor-based OA models systematically underrepresent the mechanical conditions that drive sex-dependent disease onset and progression [109,110].

Perfusion bioreactors contribute an additional dimension by introducing controlled fluid flow and shear stresses [111], which regulate nutrient transport and activate mechanotransduction cascades such as MAPK/ERK and Wnt/β-catenin signaling [112,113,114]. Similarly, cyclic hydrostatic pressure enhances chondrogenic markers (including collagen II, proteoglycans, and SOX9) via TGF-β/SMAD and IGF-1/PI3K/AKT pathways [115,116]. Yet these studies typically use loading magnitudes and duty cycles that do not reflect sex-dependent mechanical environments, such as differences in cartilage thickness, gait patterns, and muscle recruitment strategies, all of which modulate how male and female joints respond to mechanical stress.

Recent technological advances address some of these gaps. High-throughput systems such as HITMACE demonstrate that physiological loading promotes matrix deposition, whereas excessive strain induces degeneration, which are findings that align with clinical evidence of sex-differential susceptibility to overload but are rarely explored in a sex-stratified framework [117]. Likewise, the modular bioreactor developed by Gamez et al., enabling simultaneous biochemical and biomechanical control, has improved maturation of osteochondral construct [118], while dual-chamber bioreactors recreate the asymmetric nutrient and flow environments of cartilage and subchondral bone [119,120], features that could be harnessed to model the distinct remodeling behaviors observed in men and women.

**Figure 5:**
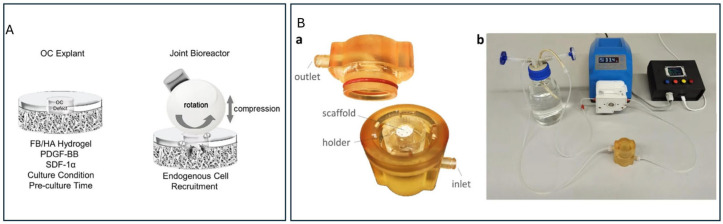
Bioreactor configurations tailored for osteochondral studies: (**A**) A four-station bioreactor inside a CO_2_ incubator (37 °C, 5% CO_2_, 85% humidity) applying a ceramic hip ball (32 mm) onto osteochondral plugs, maintaining a constant 0.4 mm displacement (10–14% of cartilage height) to ensure full contact with hydrogel and cartilage. Reprinted from Ref. [121]. (**B**) (**a**) 3D printed bioreactor chamber showing top and bottom parts with scaffold holder; (**b**) overview of main components: culture chamber, perfusion unit, and control unit. Reprinted from Ref. [122].

Representative systems include dedicated mechanical activators (Figure 5A) [121] and hybrid setups integrating bidirectional perfusion with pulsed electromagnetic fields to enhance scaffold maturation (Figure 5B) [122]. These platforms illustrate a broader shift toward combining multiple stimuli in a single device. However, their application remains predominantly sex-neutral. Incorporating sex-identified donor cells, physiologically relevant hormone concentrations, and receptor-level outcomes (ERα, ERβ, AR) would enable these bioreactors to capture how mechanical forces and endocrine cues jointly shape cartilage and bone behavior in a sex-dependent manner.

### 3.4. Microfluidic Approaches to Osteochondral Modeling

Microfluidic technologies offer unprecedented spatial and temporal control; however, their current implementation in OA research largely reproduces sex-neutral paradigms at higher resolution. Unlike bulk bioreactor systems, microfluidic platforms permit fine control of local shear stresses, molecular gradients, and compartmentalized tissue interfaces, features that can be leveraged to reproduce sex-dependent differences in nutrient transport, inflammatory signaling, and receptor-mediated responses at microscale resolution [123,124,125].

Within these devices, laminar flow enables the generation of physiologically relevant shear stress profiles that modulate cartilage and bone homeostasis [126,127,128,129]. Because shear sensitivity varies with hormonal status and sex-specific receptor expression, microfluidic environments offer a powerful means of studying how estradiol, testosterone, and their downstream pathways (ERα, ERβ, AR) shape cell–matrix interactions under controlled fluidic regimes. By tuning flow rates and perfusion routes, these systems can reproduce the asymmetric transport patterns associated with sex-dependent differences in synovial fluid viscosity, joint capsule compliance, and inflammatory burden [130].

Mechanically active microfluidic platforms further expand these capabilities. Devices incorporating deformable PDMS membranes allow cyclic compression at frequencies mimicking daily physiological activity [131]. These dynamics can be coupled with sex-specific biochemical inputs, such as peri-menopausal estradiol levels or age-related androgen decline, to test how hormonal context modulates mechanotransduction. Because women and men differ in chondrocyte mechanosensitivity, integrin signaling, and matrix turnover, these microactuated systems offer a scalable route for dissecting the interplay between sex hormones and mechanical cues with subcellular resolution [132].

**Figure 6:**
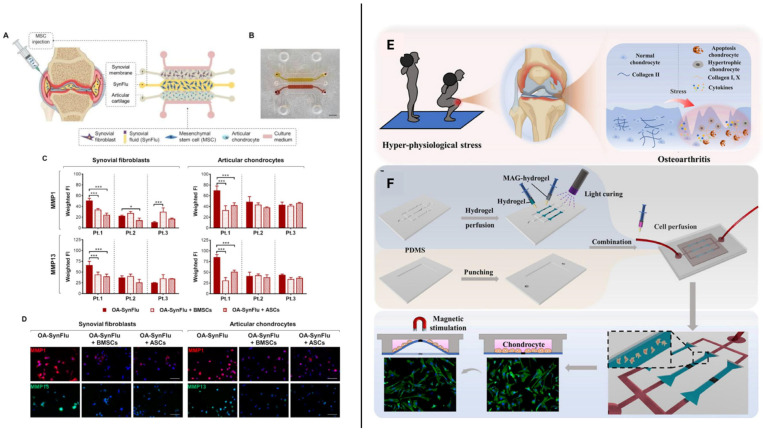
Microfluidic joint-on-a-chip models capturing key OA mechanisms. (**A**) Schematic of an OA knee joint with an injectable MSC-based therapy, showing the reproduction of each component in the chip; (**B**) Micrograph of the device with channels for the synovial (yellow) and chondral (red) compartments. Scale bar: 2 mm; (**C**) Quantification of MMP1 and MMP13 in patient-specific joint-on-a-chip models, untreated or treated with BMSCs or ASCs (n = 3; significance indicated as * *p* < 0.05, *** *p* < 0.001); (**D**) Representative staining of MMP1 (red), MMP13 (green), and nuclei (blue). Scale bars: 100 μm—(**A**–**D**) reprinted from Ref. [137]. Copyright 2024 Elsevier. (**E**) Schematic representation of inflammatory changes in the knee joint under hyper-physiological stress; (**F**) Overview of chip assembly and cell perfusion process—(**E**,**F**) Reprinted from Ref. [138].

The emergence of Joint-on-a-Chip (JOC) systems has further broadened the scope of sex-aware investigation. By integrating chondrocytes, osteoblasts, synoviocytes, and immune cells within interconnected microchambers [81,133], these platforms support the reconstruction of osteochondral crosstalk under dynamic flow while enabling precise manipulation of sex-specific variables [134,135]. Recent osteochondral organ-on-chip systems reported sex-stratified responses to inflammatory stimuli, with divergent matrix remodeling and mechanical properties [136]. Overall, JOC systems illustrate how microfluidic platforms can model key pathological processes relevant to OA (Figure 6). These devices enable the compartmentalized reconstruction of synovial and chondral tissues (Figure 6A,B), providing controlled environments for evaluating inflammatory signaling, matrix degradation, and cell–cell crosstalk. For example, quantification of matrix metalloproteinases (MMPs) 1 and 13, i.e., central mediators of OA-related cartilage catabolism, is shown in patient-specific chips untreated or treated with bone-marrow–derived mesenchymal stromal cells (BMSCs) or adipose-derived stromal cells (ASCs) (Figure 6C). Immunostaining further confirms localized MMP activation and inflammation-driven matrix remodeling (Figure 6D) [137]. Additional models reproduce hyperphysiological mechanical stress and synovial infiltration (Figure 6E,F), demonstrating that such systems can also provide a controlled environment for quantifying how sex-dependent paracrine networks regulate the activity of MMPs, cytokines, and matrix components during the onset of OA during OA onset [138].

**Figure 7:**
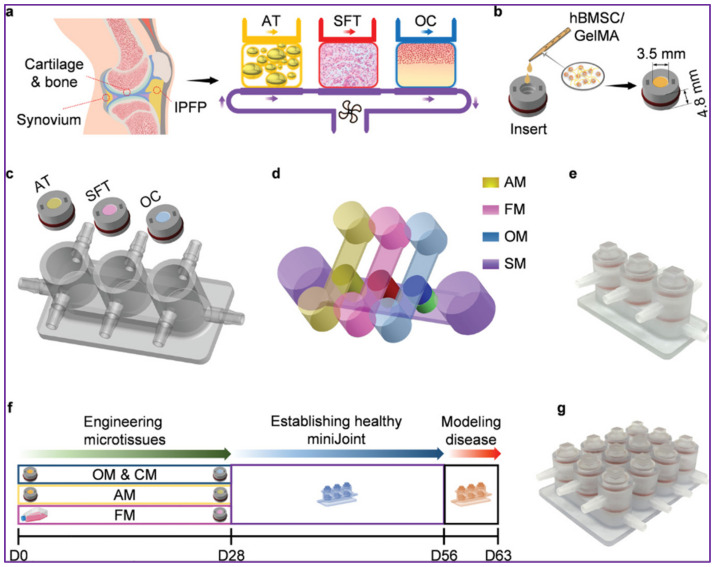
Microfluidic bioreactors advancing osteochondral research: (**a**) The miniJoint chip includes engineered adipose (AT), synovial-like fibrous (SFT), and osteochondral (OC) tissues to mimic native knee structures, with crosstalk via diffusion or fluidic flow. (**b**) Microtissues formed by photo-crosslinking hBMSC-laden GelMA in 3D printed inserts. (**c**,**d**) Chip assembled by integrating differentiated microtissues into a 3D-printed chamber (**c**) and perfusing tissue-specific media (AM, FM, OM) on top with a shared synovial-like medium (SM) at the bottom (**d**). (**e**) Photograph of the assembled miniJoint chip. (**f**) Timeline of miniJoint culture and joint disease modeling up to day 63. (**g**) High yield miniJoint producing four replicates of each microtissue. Reprinted with permission from Ref. [140].

Building on this foundation, hybrid microfluidic–bioreactor platforms have begun to reproduce multi-tissue interactions and chronic degenerative trajectories that align more closely with human OA physiology [139,140,141]. A representative example of these hybrid approaches is the “miniJoint” system developed by Li et al., which integrates 3D bioprinting, stromal cell differentiation, and microfluidic perfusion within a mechanically tunable chamber (Figure 7) [142]. In this platform, adipose tissue (AT), synovial-like fibrous tissue (SFT), and osteochondral (OC) microtissues are engineered in parallel and positioned within dedicated compartments to reproduce the multi-tissue organization and crosstalk of the native knee joint (Figure 7a). Human bone-marrow–derived mesenchymal stromal cells (hBMSCs) are photo-crosslinked within GelMA (Gelatin Methacryloyl) to generate the microtissue units (Figure 7b), which are subsequently assembled into a 3D-printed chamber and perfused with tissue-specific culture media (adipose medium (AM), fibrous medium (FM), and osteogenic medium (OM)) in combination with a shared synovial-like medium (SM) to emulate coordinated biochemical signaling (Figure 7c,d). The assembled miniJoint device (Figure 7e) supports long-term culture and progressive modeling of OA-like degeneration up to 63 days (Figure 7f), while enabling parallel production of multiple tissue replicates for high-throughput analyses (Figure 7g). Together, these capabilities demonstrate how microfluidic perfusion, 3D tissue engineering, and compartment-specific media can recreate coordinated biochemical signaling across adipose, synovial, and osteochondral compartments [142]. Although not originally conceived for sex-specific modeling, most joint-on-chip systems implicitly assume that inflammatory signaling, matrix turnover, and mechanosensitivity are sex-invariant, thereby limiting their capacity to explain divergent disease trajectories.

A distinctive advantage of microfluidic platforms in the context of sex differences lies in their capacity for high-resolution monitoring. Real-time imaging enables dynamic assessment of ERα/ERβ/AR localization, mitochondrial stress, or cytokine release under sex-tailored chemical and mechanical conditions, features that are difficult to achieve in larger-scale systems [143,144]. Moreover, the reduced reagent volumes allow systematic investigation of physiologically relevant hormone fluctuations, such as low-amplitude estradiol oscillations or pulsatile release patterns, without incurring the cost or variability associated with macroscale culture.

As summarized in Table 2, bioreactors and microfluidic devices offer complementary advantages. Whereas bioreactors excel in reproducing macroscale loading patterns, microfluidics provide fine control over biochemical gradients, fluid mechanics, and cellular interactions. Integrating these strengths represents a promising route toward next-generation OA models capable of capturing the multidimensional and sex-specific complexity of joint degeneration with both physiological fidelity and mechanistic depth.

## 4. Limitations and Solutions in Addressing Sex Differences

### 4.1. Limitations of Current Neutral-Sex OA Models

Despite significant technological progress, most in vitro OA models remain conceptually anchored to sex-neutral assumptions, limiting their explanatory and predictive power. Across 2D, 3D, bioreactor, and microfluidic systems, the primary limitation is not the absence of technology, but the persistence of a sex-neutral design paradigm that treats hormonal and biomechanical differences as optional refinements rather than foundational requirements.

Within this paradigm, scaffold-based systems exemplify how material design choices can propagate sex-neutral assumptions across experimental models. Scaffold stiffness is commonly selected to match average tissue mechanics, overlooking sex-dependent differences in mechanosensitivity that modulate chondrocyte and osteoblast responses to identical loading conditions [145]. Similarly, scaffold degradation profiles are rarely aligned with the biological timescales of OA progression, which differ between men and women due to endocrine aging and metabolic changes [146]. Finally, hormone delivery (when implemented) is typically decoupled from material behavior, preventing coordinated regulation of matrix remodeling, receptor activation, and mechanical adaptation [147].

Table 3 summarize the principal advantages and limitations of current in vitro models.

Two- and three-dimensional cultures, although widely used, provide limited mechanobiological relevance because they lack estrogen- and androgen-regulated microenvironments and cannot simulate the dynamic receptor-level responses (ERα, ERβ, AR) that underpin sex-dependent cartilage and bone behavior [148]. As a result, divergent responses to inflammatory cytokines, oxidative stress, or chondrogenic stimuli remain difficult to interpret in a sex-specific framework.

Bioreactors offer enhanced physiological relevance through dynamic loading, but most platforms apply standardized compression schemes that do not reflect the distinct kinematic patterns documented in men and women [149]. Differences in valgus alignment, muscle recruitment strategies, and load distribution (especially the higher lateral force transmission typically observed in women) are major determinants of OA susceptibility but remain absent in current designs [150]. This omission limits the capacity of bioreactors to model how sex-dependent gait mechanics translate into distinct strain fields, mechanosensitive responses, and patterns of subchondral remodeling [151].

Microfluidic Joint-on-a-Chip systems have expanded the capacity to model osteochondral crosstalk with high spatiotemporal resolution [152,153,154]. However, most devices rely on uniform perfusion schemes and lack hormone-tunable gradients, thereby constraining the study of sex-dependent inflammatory signaling, matrix turnover, and tissue–tissue interactions [131,155,156]. The absence of receptor-level endpoints further restricts the quantification of how estradiol or testosterone modulate local responses under shear stress or micro-scale compression [157].

Across all model types, the most persistent limitation is the lack of integrated sex-aware parameters: physiological hormone dynamics, sex-dependent loading paradigms, and molecular endpoints capable of resolving male–female differences [158]. While Table 3 summarizes the general advantages and limitations of current in vitro platforms, these characteristics do not explicitly capture their sex-specific shortcomings. To address this gap, Table 4 compares the main in vitro OA models according to their reproduced physiological features and their specific limitations in modeling male–female differences. As summarized in Table 4, sex bias in current in vitro OA models arises at multiple levels, including biological inputs, material and mechanical design, and the absence of sex-resolved molecular endpoints. Until these components are systematically incorporated, in vitro systems will continue to reproduce only sex-neutral averages, masking the mechanisms that drive the unequal burden of OA across the lifespan.

### 4.2. Why Are Effective Sex-Specific In Vitro Models Still Missing?

Despite clear biological rationale and growing interest, effective sex-specific in vitro OA models remain rare. This gap reflects a set of unresolved challenges at the biological, engineering, and translational levels.

While technical challenges exist, the scarcity of sex-specific OA models primarily reflects conceptual inertia: most platforms are optimized for experimental control rather than physiological validity across sexes. Estrogen, progesterone, and androgens fluctuate with age, as well as across distinct sex-specific temporal scales, including predominantly circadian (24 h) hormonal rhythms in men and menstrual cycle–dependent (~monthly) variations in women, menopausal status and metabolic conditions [159]. Replicating these temporally regulated variations would require delivery systems capable of generating pulsatile, cyclic, or gradient-based hormonal profiles with high temporal resolution. Current perfusion platforms primarily support steady-state exposure and therefore fail to capture hormonal dynamics that critically shape sex-specific cellular responses [160].

Reconstructing the osteochondral unit introduces additional complexity. The native interface exhibits marked spatial heterogeneity and functional coupling between cartilage, subchondral bone, and synovium, demanding biomaterials with distinct yet mechanically integrated properties [161]. These challenges intensify under dynamic flow, where maintaining structural integrity and biochemical identity becomes difficult. Moreover, co-culturing osteoblasts, chondrocytes, synoviocytes, and immune cells require stringent control of nutrients and oxygen gradients tailored to each cell population, i.e., a requirement that current microfluidic and bioreactor systems only partially meet [162].

Cellular variability further complicates model standardization. While iPSCs and MSCs support patient-specific platforms, they introduce donor-to-donor heterogeneity linked to genetic background, epigenetic memory, and previous inflammatory or metabolic exposures [163]. Preserving stable sex-specific phenotypes during expansion and differentiation is particularly challenging in cartilage systems, where maintaining chondrogenic identity is already difficult even before incorporating hormonal variables [164]. The lack of validated sex-stratified reference cell lines remains a major barrier to reproducibility.

Accurately mimicking sex-dependent joint biomechanics poses additional engineering constraints. Reproducing these differences would require actuation systems capable of applying compression, shear, and torsion simultaneously [165], yet most platforms lack such multiaxial control. Integrating complex loading regimens into microfluidic environments introduces risks, including compromised sterility, altered flow profiles, and reduced tissue viability, that further limit the implementation of sex-specific mechanical inputs [166].

Finally, translational progress is slowed by regulatory uncertainties. Hormone-loaded biomaterials designed to emulate sex-specific environments often display nonlinear release kinetics and variable degradation rates, hindering reproducibility and long-term stability [167]. Standardizing assays is difficult when physiological fluctuations introduce variability incompatible with existing reference frameworks [168].

The absence of explicit guidelines for validating in vitro models that incorporate sex as a biological variable creates additional uncertainty regarding acceptable endpoints, quality thresholds, and performance metrics [169]. Developing shared protocols (spanning hormone dosing schemes, multiaxial biomechanical inputs, cell sourcing strategies, and quality-control criteria) will be essential for transitioning sex-specific OA platforms from proof-of-concept systems to robust, translationally viable preclinical systems.

### 4.3. Toward Sex-Specific Innovations in OA Models

Transforming current OA platforms into sex-specific systems does not require new technologies but rather a conceptual shift in how existing tools are configured. Introducing sex-aware variables (hormonal, mechanical, cellular, and molecular) into established in vitro models can substantially enhance their translational relevance.

Two-dimensional cultures offer a straightforward entry point by allowing the use of sex-identified donor cells, physiological hormone levels, and controlled exposure schemes that mimic peri-menopausal estrogen decline or age-related androgen reduction [170,171]. These setups provide rapid insights into sex-dependent differences in stress responses, apoptosis, autophagy, and extracellular matrix turnover [172].

Three-dimensional osteochondral scaffolds are particularly well positioned for sex-specific modeling because they provide a structural framework for integrating endocrine signals, mechanical cues, and receptor-level readouts [173]. Embedding sex-stratified cells within engineered matrix architectures allows systematic interrogation of ERα/ERβ/AR activity under controlled microenvironmental conditions. This approach enables direct assessment of how sex influences key processes such as MSC differentiation, mineralization kinetics, and susceptibility to inflammatory degradation, which remain largely unexplored in the current literature.

Bioreactors, which are widely used for applying dynamic loading, can be readily adapted for sex-specific mechanobiology by calibrating compression, shear, or hydrostatic cycles to male–female gait characteristics and muscle-driven load profiles [170]. Perfusion with hormone-conditioned media further allows the evaluation of how mechanical and endocrine stimuli interact to regulate mechanotransduction pathways such as NF-κB, Wnt/β-catenin, and TGF-β/SMAD [174].

Microfluidic Joint-on-a-Chip platforms offer unmatched capacity for fine-tuned manipulation of sex-specific variables. Parallel-channel configurations can model male and female microenvironments simultaneously, while compartment-specific perfusion permits the delivery of tailored estrogen, progesterone, or androgen concentrations [84]. Integration of biosensors for cytokines, oxygen, pH, or cartilage degradation markers (i.e., CTX-II) provides real-time quantification of divergent inflammatory and metabolic responses [175]. By enabling side-by-side comparison of male- and female-derived tissues under identical conditions, these platforms can uncover subtle mechanistic differences that would be obscured in larger-scale systems.

Finally, to ensure clinical relevance, regulatory frameworks must evolve to mandate sex-balanced preclinical assessments. Establishing standardized benchmarks for sex-aware mechanical loading, hormone dosing, and molecular endpoints will support reproducibility and accelerate integration into precision-medicine pipelines [10].

**Figure 8:**
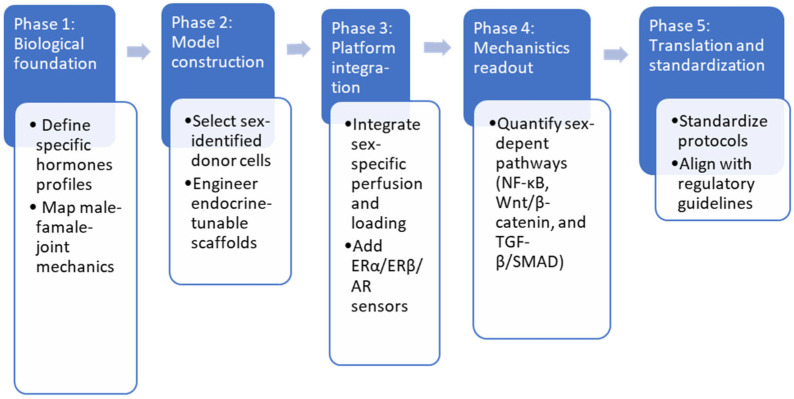
Roadmap for the development of next-generation sex-specific OA models through a five-phase progression: Phase 1 establishes the biological foundations by defining hormone profiles and mapping sex-dependent joint mechanics. Phase 2 focuses on constructing the model using sex-identified donor cells and endocrine-tunable scaffolds. Phase 3 integrates platform-level components, including sex-specific perfusion schemes, mechanical loading, and receptor-based sensors (ERα, ERβ, AR). Phase 4 centers on mechanistic readouts, quantifying key pathways such as NF-κB, Wnt/β-catenin, and TGF-β/SMAD under sex-tailored conditions. Phase 5 advances toward translation by standardizing protocols and aligning them with emerging regulatory guidelines. Together, these phases provide a structured path for converting existing technologies into physiologically relevant sex-specific osteoarthritis models.

To operationalize the transition toward sex-specific in vitro systems, the conceptual principles outlined above can be organized into a structured roadmap (Figure 8). This framework delineates the progressive steps required to integrate sex-aware variables across biological setup, model construction, platform engineering, mechanistic readouts, and translational standardization. By advancing through these phases, current technologies can gradually incorporate hormone dynamics, sex-dependent biomechanics, and receptor-level signaling, ultimately enabling more predictive and physiologically coherent osteoarthritis models.

## 5. Conclusions

Osteoarthritis cannot be understood, modeled, or treated effectively without explicitly incorporating sex as a fundamental biological dimension. The evidence consolidated in this review demonstrates that sex differences are not secondary modifiers of joint degeneration but primary drivers of distinct mechanobiological, endocrine, and inflammatory trajectories. Yet most in vitro systems still operate under sex-neutral assumptions that obscure clinically relevant divergence between male and female tissues.

The technological tools needed to overcome this gap already exist. Advanced multiphasic scaffolds, dynamic bioreactors, and microfluidic JOC platforms are fully capable of integrating sex-specific variables; however, they are rarely configured within a framework that embeds hormonal dynamics, sex-dependent biomechanics, and receptor-level signaling as design principles rather than post hoc additions. Progress, therefore, hinges not on inventing new devices but on redefining how existing biomaterial-based platforms are configured, validated, and interpreted.

Sex-aware modeling is not an optional refinement but a prerequisite for mechanistic validity in osteoarthritis research. Rather than adding hormones as isolated variables, sex-specific modeling requires coupling endocrine context with material properties and mechanical boundary conditions from the outset. In particular, scaffold stiffness and degradation kinetics should be interpreted relative to sex-dependent mechanosensitivity and disease timing, while mechanical loading schemes should reflect known differences in joint kinematics. Emphasizing receptor-level and pathway-resolved readouts would further enable a shift from descriptive comparisons toward mechanistic explanations of sex-divergent OA trajectories. Without this conceptual shift, increasingly complex in vitro models will continue to generate sex-averaged data that fail to explain, predict, or stratify osteoarthritis across the human population.

## Figures and Tables

**Table 1:** Roles of estrogen and testosterone in OA in women and men. It highlights how sex hormones regulate joint homeostasis through distinct receptor-mediated pathways. These mechanisms are rarely co-implemented in current in vitro OA models, despite being central to sex-specific disease trajectories. MAPK: Mitogen-activated protein kinase, ERK: Extracellular signal-regulated kinase, NF-kB: Nuclear Factor kappa-light-chain-enhancer of activated B cells, IL-1β: Interleukin-1 beta, TNF-α: Tumor Necrosis Factor-alpha, ER-α: Estrogen receptor α, ER-β: Estrogen receptor β, AR: Androgen receptor, Wnt: Wingless-related integration site.

Hormone	Role in Women	Role in Men	Pathways
Estrogen (E2)	Regulates osteoclastic activity; promotes cartilage stability. Post-menopausal reduction is associated with accelerated joint degeneration.	Lower direct impact; Indirect influence via testosterone aromatization	ER-α—Regulation of osteoblast and osteoclast activity (MAPK, ERK); ER-β—Modulation of inflammatory cytokine production in chondrocytes and synovial tissue (NF-kB, IL-1β, TNF-α);
Testosterone	Limited direct impact; low levels may contribute to post-menopausal bone loss indirectly via reduced aromatization to estradiol.	Regulates bone turnover and cartilage density; low levels may increase the risk of OA	AR—Promotes osteoblast activity and influences cartilage matrix synthesis. Wnt—Involvement in chondrocyte differentiation.

**Table 2:** Advantages and trade-offs of bioreactors and microfluidic devices in osteoarthritis research.

Feature	Bioreactors	Microfluidic Devices
Scale	Macro-scale, suitable for larger tissue constructs	Micro-scale, ideal for cellular and microscale tissue environments
Mechanical stimulation	Compression, tension, cyclic hydrostatic pressure	Precise shear stress and localized compression
Fluid flow	Large-volume perfusion and long-term medium circulation	Microscale, controlled perfusion, and synovial-like flow
Real-time monitoring	Often requires external sensors or imaging setups	Built-in optical access enabling continuous high-resolution imaging
Relevance to OA studies	Effective for simulating joint-level loading and long-term degeneration	Suited for studying inflammatory signaling, mechanotransduction, and drug screening
Cost and complexity	Higher cost and more complex instrumentation	Lower cost, simpler setup, and compatible with high-throughput experiments

**Table 3:** Advantages and disadvantages of current in vitro models.

Model Type	Advantages	Disadvantages
2D/3D in vitro models	Cost-effective; suitable for high-throughput screening	Limited reproduction of in vivo complexity; inadequate simulation of joint-specific mechanical forces
Bioreactors	Enable dynamic compression, shear, and hydrostatic pressure; support tissue maturation	Difficulty reproducing multiaxial joint forces; limited scalability and reproducibility
Microfluidic JOC technologies	High precision in controlling fluid flow and shear stress; real-time monitoring	Often restricted to single-tissue compartments; incomplete reproduction of synovial flow and multiscale mechanics
Microfluidic Bioreactors	Precise control of nutrient/oxygen gradients; real-time monitoring; support multilayer constructs	Technically demanding; still unable to reproduce all disease aspects

**Table 4:** Sex-related limitations of current in vitro osteoarthritis models. Comparison of major in vitro platforms highlighting how sex-specific variables are omitted at the level of biological inputs, mechanical design, and molecular readouts.

In Vitro OA Model	Main Reproduced Features	Sex-Specific Variables Not Captured
2D culture (chondrocytes, osteoblasts, synoviocytes)	Isolated inflammatory signaling and response to exogenous hormones [19].	Donor sex rarely reported; hormones addition does not reproduce physiological fluctuations (menopause, hypogonadism).
3D scaffold (porous, multilayered)	ECM maintenance and osteochondral-like architecture [92,100,101,102,103,104].	Donors rarely stratified by sex; limited assessment of estrogen/testosterone effects on MSC differentiation, mineralization, or inflammation.
Bioreactors	Dynamic compression, shear stress, hydrostatic pressure promoting ECM deposition and tissue maturation [119,120,121,122,123,124].	Absence of sex-specific hormonal profiles; loading schemes not calibrated to sex-dependent biomechanics (i.e., Q-angle, force distribution).
Microfluidic JOC	Dynamic co-cultures of cartilage, bone, and immune cells with controlled shear [136,137,138,139,140].	No incorporation of estrogen/testosterone fluctuations; absence of sex-dependent hormonal gradients.
Microfluidic bioreactors	High control of nutrients/oxygen; real-time monitoring; multilayer constructs [141,142,143,144].	No perfusion with physiological hormone levels; absence of ERα, ERβ, and AR endpoints; loading not calibrated to male/female biomechanics.

## Data Availability

No new data were created or analyzed in this study. Data sharing is not applicable to this article.

## Abbreviations

The following abbreviations are used in this manuscript:

2D	Two-dimensional
3D	Three-dimensional
AKT	Protein Kinase B
AR	Androgen receptor
BCP	Biphasic calcium phosphate
BMD	Bone mineral density
BMP	Bone Morphogenetic Protein
CAD	Computer-Aided Design
COX-2	Cyclooxygenase-2
CTX	C-terminal telopeptide of collagen
ECM	Extracellular matrix
ER	Estrogen receptors
ERK	Extracellular signal-regulated kinase
HRT	Hormone replacement therapy
IL-1β	Interleukin-1 beta
IL-6	Interleukin-6
IGF-1	Insulin-like Growth Factor 1
iPSC	Induced Pluripotent Stem Cells
JOC	Joint-on-a-chip
MMPs	Matrix metalloproteases
MSCs	Mesenchymal stem cells
MAPK	Mitogen-activated protein kinase
NF-kB	Nuclear Factor kappa-light-chain-enhancer of activated B cells
NHANES	National Health and Nutrition Examination Survey
NSAIDs	Nonsteroidal Anti-Inflammatory Drugs
OA	Osteoarthritis
PCL	Poly(ε-caprolactone)
PDMS	Polydimethylsiloxane
PI3K	Phosphatidylinositol 3-kinase
PINP	Pro-collagen type I N-terminal pro-peptide
PLA	Polylactic acid
PLGA	Poly(lactide-co-glycolide)
SERMs	Selective estrogen receptor modulators
SOX9	SRY-box transcription factor 9
TGF-β	Transforming Growth Factor-beta
TNF-α	Tumor Necrosis Factor-alpha
Wnt	Wingless-related integration site
